# Dose optimization in CBCT in dentistry: a survey among EADMFR members

**DOI:** 10.1093/dmfr/twaf066

**Published:** 2025-09-24

**Authors:** Shayan Fakhtei, Reinier Hoogeveen, Erwin Berkhout

**Affiliations:** Department of Oral Radiology and Digital Dentistry, Academic Centre for Dentistry Amsterdam (ACTA), University of Amsterdam and VU Amsterdam, Amsterdam 1182 DB, The Netherlands; Department of Oral Radiology and Digital Dentistry, Academic Centre for Dentistry Amsterdam (ACTA), University of Amsterdam and VU Amsterdam, Amsterdam 1182 DB, The Netherlands; Department of Oral Radiology and Digital Dentistry, Academic Centre for Dentistry Amsterdam (ACTA), University of Amsterdam and VU Amsterdam, Amsterdam 1182 DB, The Netherlands

**Keywords:** cone beam CT, survey, radiation protection, ALADAIP, optimization

## Abstract

**Objectives:**

The aim of this study is to provide an overview of current cone beam CT (CBCT) practices in European dental clinics.

**Methods:**

From February to May 2023, 186 European EADMFR members were invited to complete an online survey. Participants were asked: (1) to provide exposure settings for 4 diagnostic scenarios; (2) if and how they adjust for age and body type; (3) whether they follow established protocols when selecting settings; and (4) if the Dose Area Product (DAP) is registered for CBCT scans.

**Results:**

A total of 59 (32%) eligible responses were received. There was a large variation in exposure parameters among clinicians within the same diagnostic scenario. Across scenarios, only voxel size and field of view (FOV) differed significantly (*P *< .05), with smaller settings being chosen for endodontic diagnostics and larger parameters for full-jaw imaging for implant planning. For young patients, most respondents reduced FOV (70%), mA (67%), and exposure time (59%). For larger patients, most increased mA (54%) and kV (52%). Written protocols were more frequently employed than manufacturer-recommended settings (75% vs 50% reported “most of the time” or “always”). More frequent usage of manufacturer-recommended settings correlated with larger FOV settings (*P < *.05). 90% reported registering the DAP.

**Conclusions:**

CBCT exposure parameter selection lacks consistency. Only FOV and voxel size were consistently adjusted for the indication. New guidelines providing guidance on the selection of CBCT settings could reduce variation between clinicians and enhance the uniformity of the quality of care.

**Advances in knowledge:**

Lack of uniformity in CBCT parameter selection highlights the need for updated, indication-oriented, and patient-specific guidelines.

## Introduction

The first guideline on the usage of cone beam CT (CBCT) was published by the European Academy of DentoMaxilloFacial Radiology (EADMFR) in 2009.^1^ This guideline contains 20 basic principles for the use of CBCT. In 2012, the European SEDENTEXCT (Safety and Efficacy of a New and Emerging Dental X-ray Modality—Cone Beam CT) project devised a comprehensive set of evidence-based guidelines.^2^ This document covers a range of topics, such as justification and referral criteria, best practices, and recommendations for dose optimization. Diagnostic reference levels (DRLs) were also discussed in the 2012 guidelines. DRLs define a Dose Area Product (DAP) that is deemed reasonable for a specific indication. Due to a lack of large-scale audits, no DRLs were proposed. Even today, the implementation of DRLs remains limited.^3^^,^^4^

These guidelines serve as a good foundation for the use of CBCT; however, the most recent literature cited dates back to 2011. Due to CBCT’s rapid evolution, certain aspects of these guidelines may be considered outdated. This is especially important when looking at low-dose protocols, as these can significantly reduce the effective dose while keeping the image quality at acceptable levels, in certain cases up to 15-fold compared to high-resolution imaging.^5^^,^^6^ The American Association of Endodontics (AAE) and American Academy of Oral and Maxillofacial Radiology (AAOMR) published a joint position statement on the usage and indication of CBCT in endodontics.^7^ The AAOMR also published a position statement on the clinical recommendations regarding the usage of CBCT in orthodontics.^8^ Both papers provide general recommendations for optimizing the effective dose but do not offer patient-specific or indication-oriented guidelines.

Additionally, there is a lack of guidelines specifying which settings should be used in different clinical scenarios. A position statement published by the DIMITRA (Dentomaxillofacial paediatric imaging: an investigation towards low-dose radiation-induced risks) project focusses on the justification, and most importantly, the optimization of CBCT usage in different clinical scenarios in paediatric dentistry.^9^ The DIMITRA paper builds upon the Image Gently initiative, aimed at improving the safe and effective imaging of paediatric patients.^10^ However, these recommendations remain open to clinicians’ interpretation. To ensure consistency of diagnostic quality and effective dose across clinical practices, it is desirable to have concrete guidelines on imaging parameters.

The paper also introduced As Low As Diagnostically Acceptable being Indication-oriented and Patient-specific (ALADAIP) as a more focussed interpretation of the As Low As Reasonably Achievable (ALARA) and As Low As Diagnostically Acceptable (ALADA) principles.^9^ ALADAIP emphasizes dose reduction through indication-oriented and patient-specific exposure optimizations. Large dose reductions have already been achieved with personalized optimization.^11^ It is therefore important that operators take these factors into consideration when taking CBCT scans.

Currently, there are various commercially available CBCT systems, not only differing in exposure parameters but also in post-processing capabilities. Manufacturers provide reference protocols for different indications to assist clinicians in selecting exposure parameters. These manufacturer-recommended protocols may be suboptimal, often prioritizing image quality over dose reduction. Some clinics have developed written protocols, in line with ALADAIP, to optimize exposure. It is currently not known what effects these protocols have on exposure parameters.

Compared to 2D imaging modalities, CBCT is significantly more expensive and generally exposes the patient to a higher radiation dose. Additionally, CBCT possesses more imaging parameters that contribute to increased radiation exposure compared to 2D imaging. This makes it crucial for CBCT operators to select proper settings for each case individually, as the effective dose can vary significantly depending on the parameters selected. Furthermore, different CBCT systems offer varying amounts of configurability. Systems with limited settings for field of view (FOV), resolution, or exposure may hinder precise adjustments needed to optimize the effective dose. Thus, practitioners must be aware of which parameters can and should be optimized to achieve their diagnostic goal, while minimizing the required effective dose.

The absence of studies providing clear guidelines on the optimization of exposure parameters for different indications can result in elevated radiation doses without clear clinical justification. There is currently limited evidence on what settings are being used in practice. Recently, a paper was published on the frequency and exposure settings of various dental universities in Europe.^12^ The article provides information on which settings are being used in a university clinic. This might, however, not be representative of what is being used in private clinics.

The aim of this study therefore is to create an inventory of current CBCT practices in Europe regarding the selection of settings in CBCT imaging for different patient groups and diagnostic questions. Specifically, this paper aims to answer the following questions:

- What CBCT settings are being used to answer specific diagnostic questions (implantology, orthodontics, and endodontics)?
- What CBCT settings are being used for patients with different physical characteristics?
- What is the effect of manufacturer-recommended settings and written protocols on exposure parameters?
- Is there a relation between the number of operators and settings utilized?
- Is the DAP being registered for CBCT scans performed in clinics?

## Methods

Required data were collected through a cross-sectional survey.

### Survey design

The survey presented 4 scenarios in the fields of orthodontics, implantology, and endodontics:

1. impaction of the upper left canine with possible resorption of the upper left lateral incisor
2. single-tooth implant planning for a lower molar
3. detection of a vertical root fracture in an upper molar
4. implant planning for a fixed bridge in the maxilla (4-6 implants).

Participants were asked to select the appropriate CBCT settings for the specific scenario and diagnostic question. Subsequently, they were asked whether they would adjust exposure parameters when presented with a younger or heavier-built patient. The final questions were regarding the usage of CBCT in dental practice. Participants were asked whether the DAP is recorded, the number of CBCT operators active in the clinic, how the selection of settings is determined, and the number of settings currently in use. A 5-point Likert scale was utilized for questions regarding the selection of settings. A full copy of the survey is available in Supplementary Material 4.

### Survey methodology

E-mail addresses of EADMFR members were obtained from the membership database. 186 European EADMFR members, selected by the Chair of the Selection Criteria Committee and Radiation Protection of the EADMFR, who were deemed to be involved with CBCT, were sent survey invitations. The survey was administered in English using Qualtrics (Provo, UT, United States). Initial invitations to participate were sent on February 13, 2023. Reminder emails were sent to non-responders and individuals who had not completed the survey at an interval of 1.5 weeks. The survey was closed on March 9, 2023. Participants who uncomfortable using Qualtrics were sent an open document version of the survey.

### Statistical evaluation

Data were statistically tested for normality using the Shapiro-Wilk test. The level of significance was set at 0.05. Data regarding the selection of CBCT settings for 4 different scenarios were not normally distributed, thus Kruskal-Wallis tests were performed. Dunn’s test for multiple comparisons with Bonferroni adjustment was used to perform post-hoc statistical analysis. Data regarding the influence of written, or manufacturer-recommended protocols, were statistically analysed using Spearman’s correlation. Data were statistically evaluated using R Statistical Software (version 4.4.2). Data cleaning and preparation were done using the R packages “dplyr,” “stringr,” and “tidyr.” Plots were generated using “ggplot2.”

### Ethical approval

The study setup was reviewed and approved by the Ethical Committee (ETC) on September 23, 2022, under protocol number 2022-97034. All participants provided informed consent prior to taking part in the survey.

## Results

**Figure 1.**
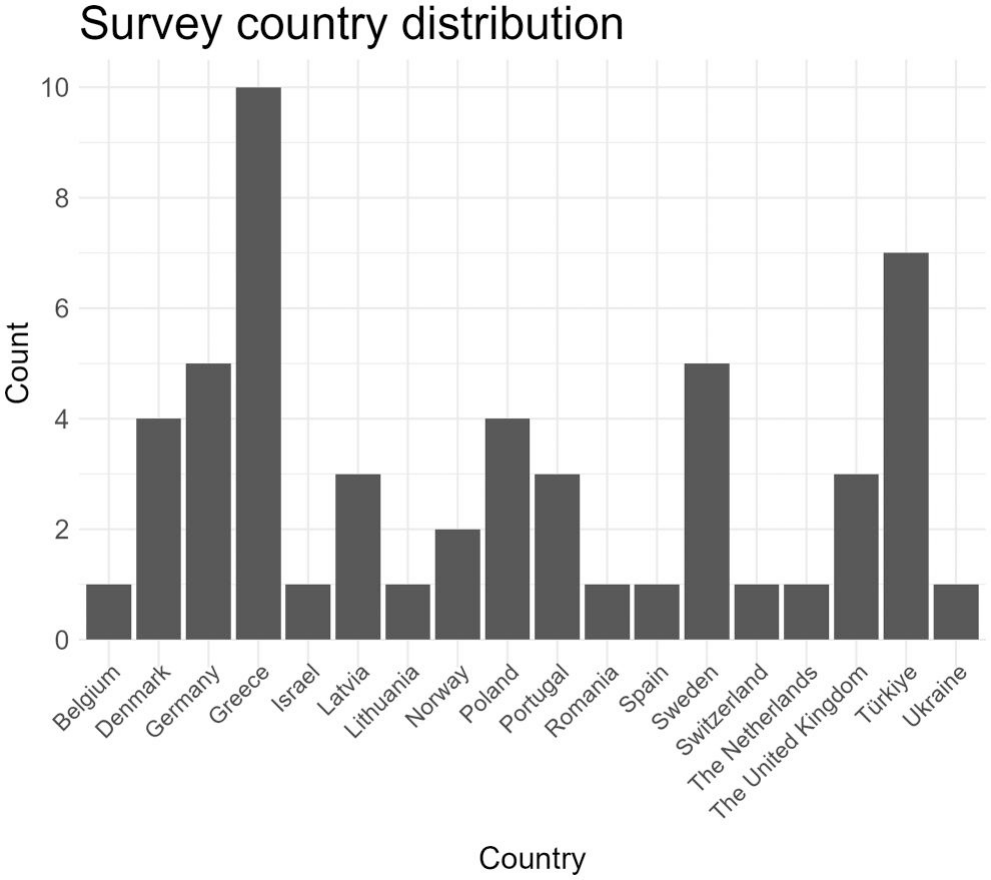
Survey participant distribution across countries.

A total of 59 (32%) fully or partially completed responses were received. Participants from the following 17 countries participated in the survey: Belgium, Denmark, Germany, Greece, Israel, Latvia, Lithuania, Norway, Poland, Portugal, Romania, Spain, Switzerland, the Netherlands, the United Kingdom, Türkiye, and Ukraine. The distribution of participants across countries is shown in Figure 1.

**Figure 2.**
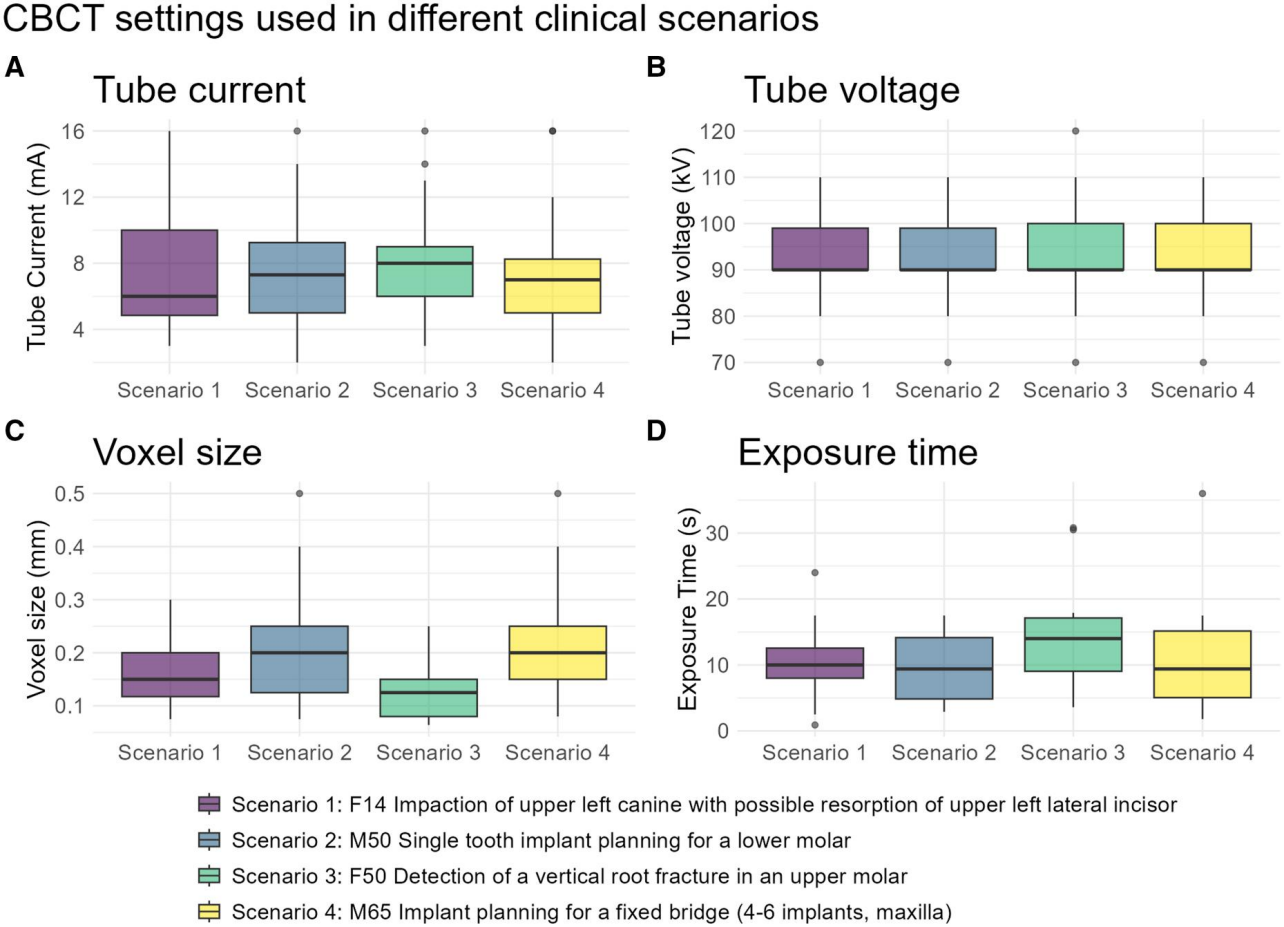
CBCT settings (A: tube current, B: tube voltage, C: voxel size, and C: exposure time) used in different clinical scenarios. Abbreviation: CBCT = cone beam CT.

Different CBCT settings used for each scenario are shown in Table 1 and Figure 2. Tube current ranged from 2.0 to 16 mA (median: 7.1 mA, IQR: 5.0-9.0 mA). Among the 80% of participants whose CBCT systems allowed adjustments, 42% adjusted the kV settings. Tube voltage ranged from 70 to 120 kV (median: 90 kV, IQR: 90-100 kV). Voxel size showed a large difference between scenarios, with sizes used ranging between 0.064 and 0.50 mm (median: 0.15 mm, IQR: 0.10-0.20 mm). Reported exposure time ranged from 0.9 to 36 s (median: 10.3 s, IQR: 6.0-15.6 s).

**Table 1.** CBCT settings used in different clinical scenarios.

	Exposure parameters									
Scenario	Tube current (mA)		Tube voltage (kV)		Exposure time (s)		Voxel size (mm)		Field of view area (cm^2^)	
	Mean (SD)	Median (IQR)	Mean (SD)	Median (IQR)	Mean (SD)	Median (IQR)	Mean (SD)	Median (IQR)	Mean (SD)	Median (IQR)
Scenario 1	7.18 (3.55)	6.00 (4.85-10.00)	92.64 (9.47)	90.00 (90.00-99.00)	10.12 (4.43)	10.00 (8.00-12.55)	0.15 (0.06)^a,c^	0.15 (0.12-0.20)	33.08 (35.26)^a^	25.00 (16.00-36.00)
Scenario 2	7.49 (3.12)	7.30 (5.00-9.25)	92.64 (9.47)	90.00 (90.00-99.00)	10.06 (4.95)	9.40 (4.85-14.14)	0.19 (0.09)^b,c^	0.20 (0.12-0.25)	26.21 (16.68)^a,b^	20.00 (16.00-25.00)
Scenario 3	7.85 (2.92)	8.00 (6.00-9.00)	94.09 (9.63)	90.00 (90.00-100.00)	13.39 (6.31)	14.00 (9.05-17.12)	0.13 (0.06)^a^	0.12 (0.08-0.15)	20.38 (8.11)^b^	16.00 (16.00-25.00)
Scenario 4	7.21 (3.13)	7.00 (5.00-8.25)	93.57 (8.82)	90.00 (90.00-100.00)	10.73 (6.67)	9.40 (5.05-15.15)	0.22 (0.09)^b^	0.20 (0.15-0.25)	60.62 (33.14)^c^	50.00 (40.00-64.00)
	*P *= .465		*P *= .767		*P *= .052		*P *< .0001		*P *< .0001	

Abbreviation: CBCT = cone beam CT.

Scenario 1: impaction of the upper left canine with possible resorption of the upper left lateral incisor; Scenario 2: single-tooth implant planning for a lower molar; Scenario 3: detection of a vertical root fracture in an upper molar; Scenario 4: implant planning for a fixed bridge in the maxilla (4-6 implants). Different superscript letters within the same column indicate statistically significant differences (*P *< .05).

**Figure 3.**
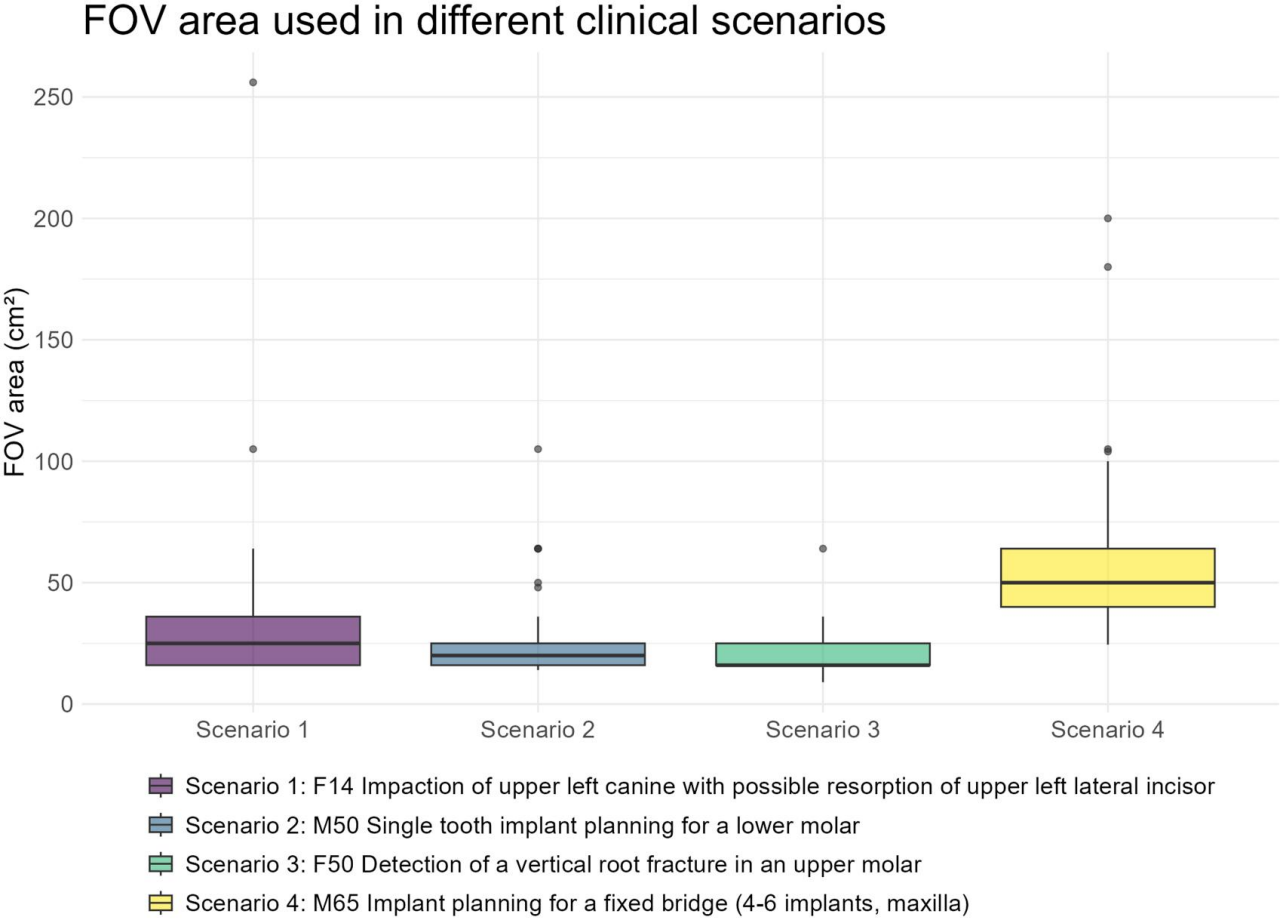
FOV area used in different clinical scenarios. Abbreviation: FOV = field of view.

Tube current, tube voltage, and exposure time did not differ significantly (*P *> .05) between the scenarios. Voxel size (Figure 2C), however, differed significantly (*P *< .0001) between the 4 scenarios (Table 1). Post-hoc analysis revealed a significant difference of voxel sizes between scenarios 1 and 4 (*P *= .0015, *Z* = 3.66), scenarios 2 and 3 (*P *= .0038, *Z* = −4.00), and between scenarios 3 and 4 (*P *< .0001, *Z* = −5.55). FOV area differed significantly (*P *< .0001) between the 4 scenarios (Figure 3; Table 1). Post-hoc statistical analysis showed a significant difference between scenario 1 and 4 (*P *< .0001, *Z* = −6.27), scenario 2 and 4 (*P *< .0001, *Z* = −7.35), scenario 3 and 4 (*P *< .0001, *Z* = −9.08), and scenario 1 and 3 (*P *= .021, *Z* = −2.91).

**Figure 4.**
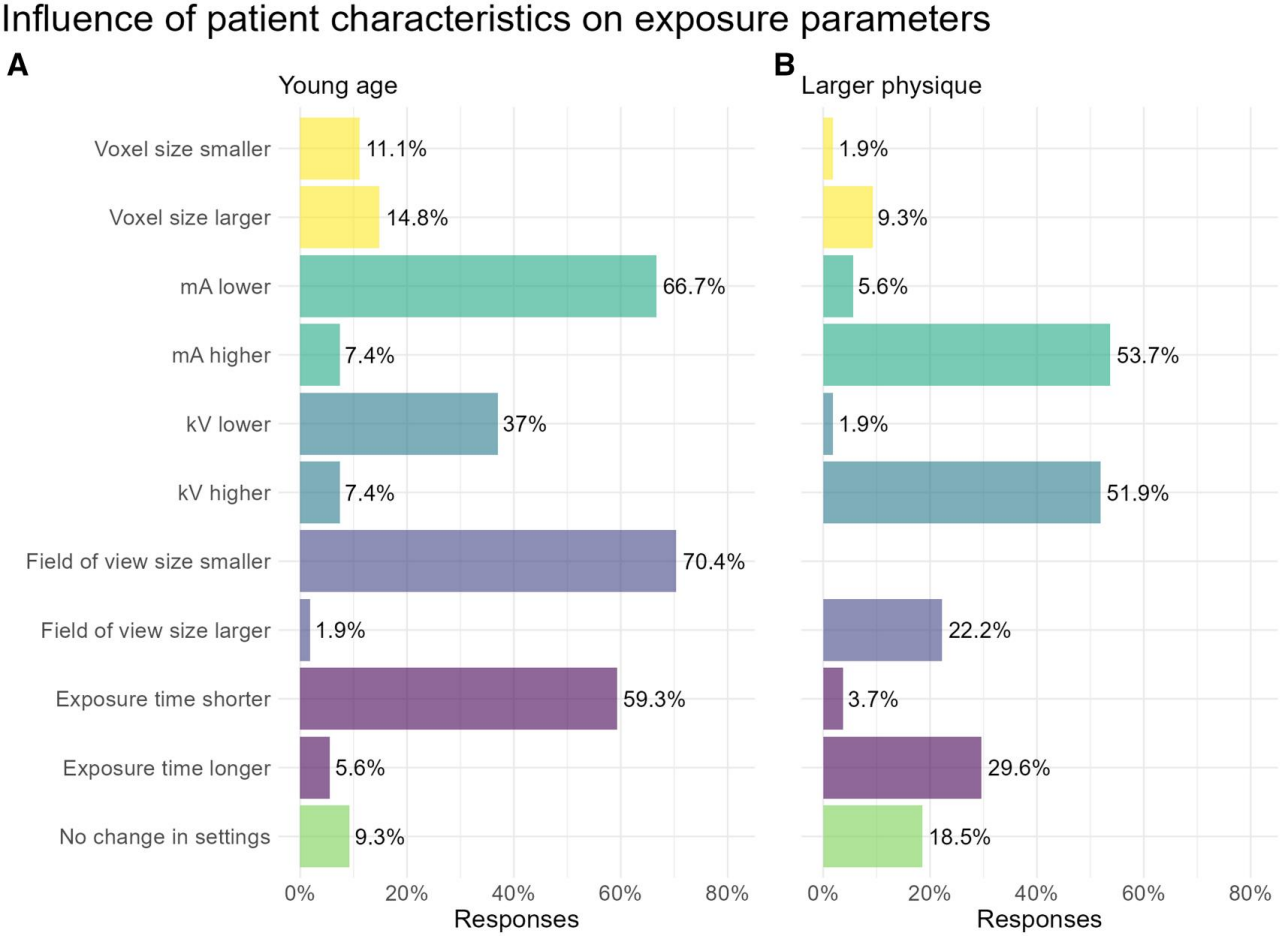
Influence of patient characteristics on exposure parameters: A: Young age; B: Larger physique.

Figure 4 shows the influence of patient characteristics on exposure parameters. Figure 4A shows the responses for a young patient. A smaller FOV was most frequently indicated (70%), followed by a lower tube current (67%), a shorter exposure time (59%), and a lower tube voltage (37%). Age did not influence the choice of exposure parameters for 9% of participants.

Figure 4B shows the responses for a patient with a larger physique. The most reported changes were an increase in the tube current (54%) and a higher tube voltage (52%). Less frequently indicated were a longer exposure time (30%) and a larger FOV (22%). For 19% of respondents, a larger physique did not influence their choice of exposure parameters.

**Figure 5.**
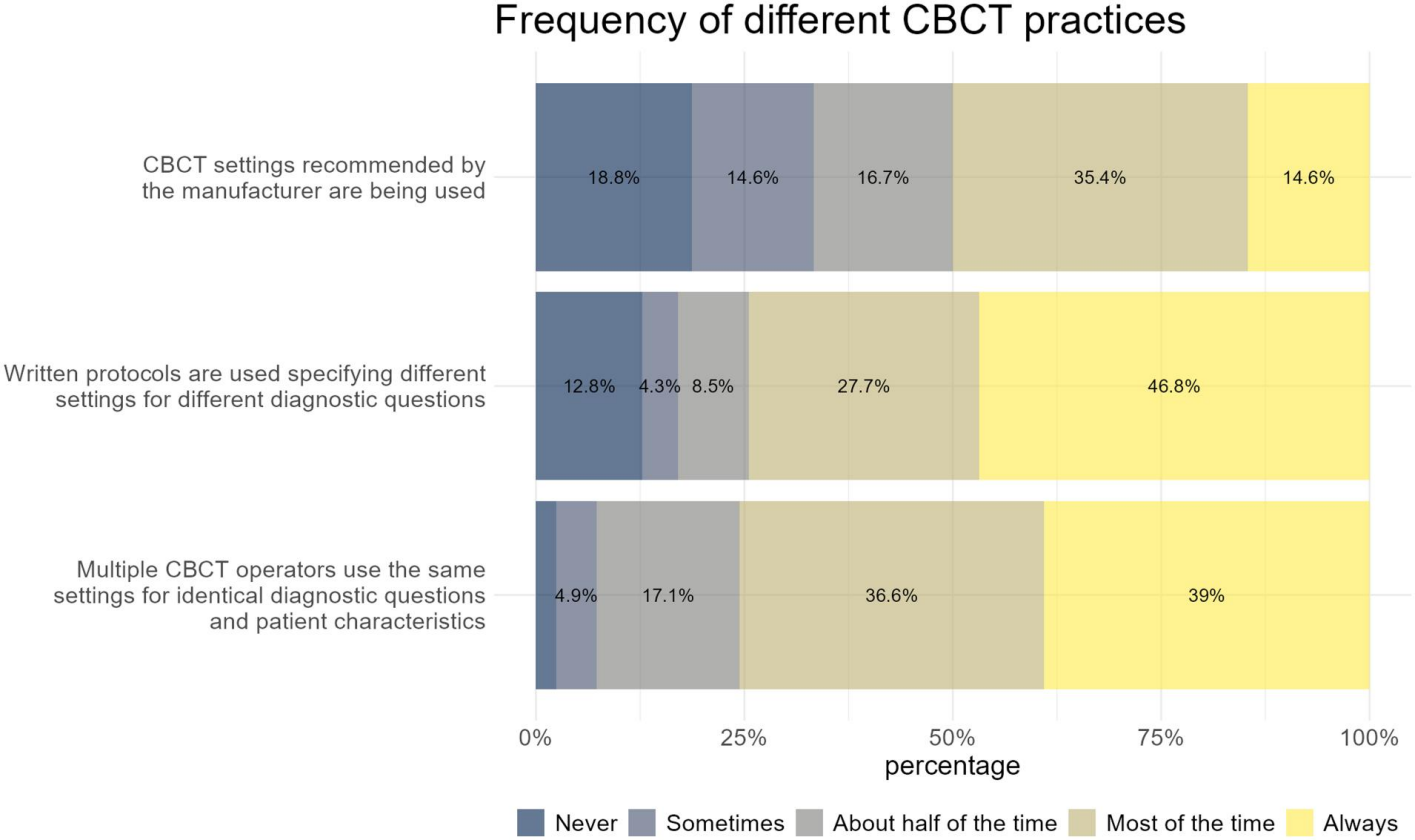
Frequency of different CBCT practices. Abbreviation: CBCT = cone beam CT.

Different CBCT practices regarding the selection of settings are shown in Figure 5. 47% of participants stated that written protocols were “always” used. 28% of the participants reported that written protocols were used “most of the time.” Furthermore, if multiple CBCT operators are present, the same settings are generally used for identical diagnostic questions, with 39% of participants indicating that identical settings are “always” used, and 37% stating that these are used “most of the time.” The usage of manufacturer-recommended settings was more variable. 33% of participants reported that manufacturer-recommended settings were “never” or “sometimes” used, while 50% of participants reported using them “most of the time” or “always.”

**Figure 6.**
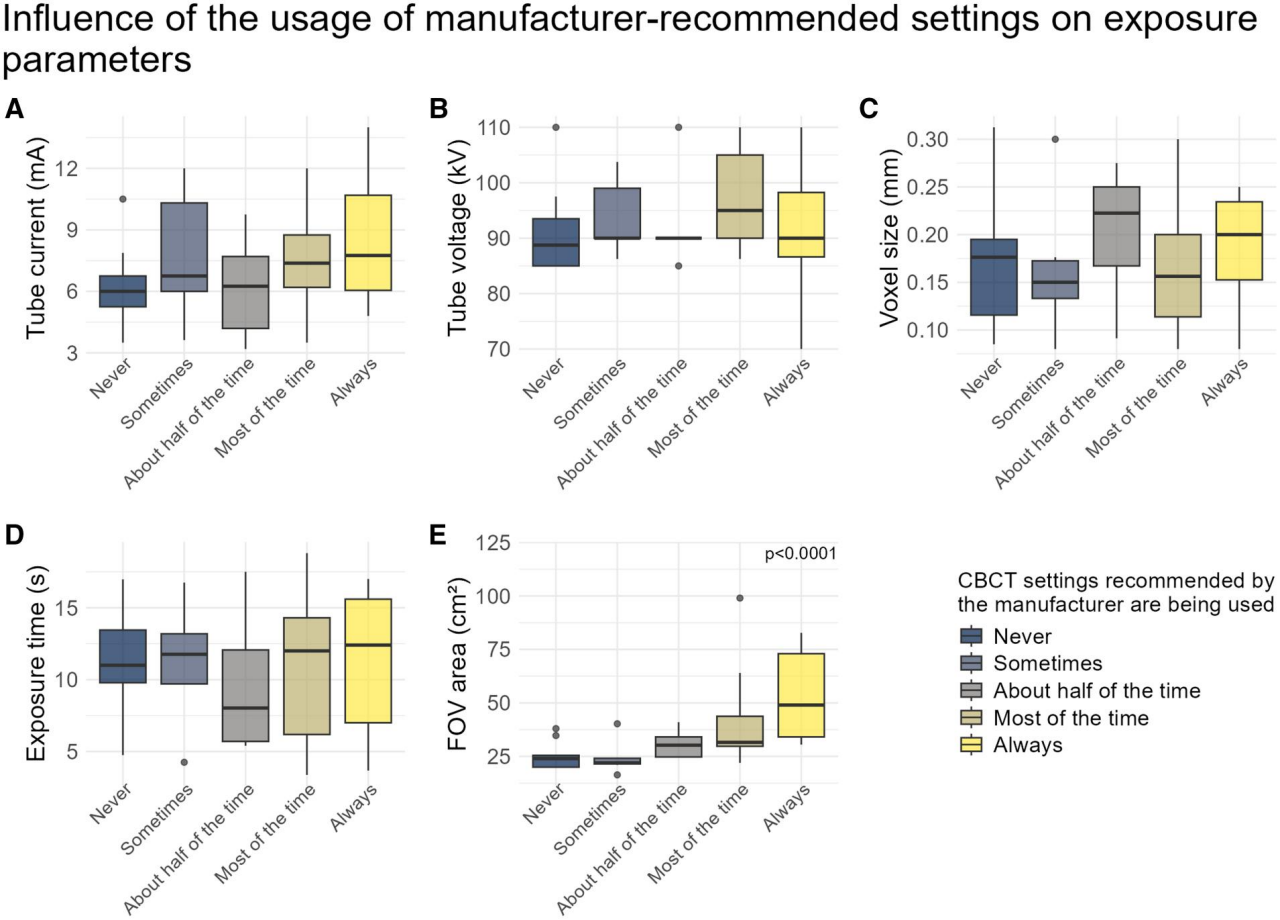
Influence of the usage of manufacturer-recommended settings on exposure parameters. For the exposure parameters, the average was taken between the 4 scenarios. Participants who did not complete all 4 scenarios were excluded. A: Tube Current (mA); B: Tube Voltage (kV); C: Voxel size (mm); D: exposure time (s) and E: Field of View area (cm^2^).

The influence of settings recommended by the manufacturer on exposure parameters is shown in Figure 6. Only the FOV area in Figure 6E differed significantly (*P *< .0001), indicating that the FOV area increased with more frequent use of manufacturer-recommended settings. The use of written protocols did not have a significant impact on chosen exposure parameters.

No significant difference was observed between the number of settings used and the number of CBCT operators active in the clinic (Supplementary Material 1).

Most participants reported registering the DAP (90%), while 10% stated the DAP was not being registered.

## Discussion

This study aimed to create an overview of current dental CBCT practices in Europe.

Among the different clinical scenarios, participants mostly adjusted the voxel size and FOV, while parameters such as tube current or exposure time were changed less often. This contrasts with existing literature, where a reduction in tube current or exposure time has shown promising results in the detection of root fractures,^13^ solitary implant planning,^14^ and the detection of impacted canines.^15^ This discrepancy suggests that operators may not be fully utilizing these parameters to optimize dose reduction, despite available evidence.

Variations in FOV align with literature, where large dose reductions were found by limiting the FOV,^16–19^ especially by reducing the FOV height.^20^ While adjusting the FOV for full-arch imaging is sometimes necessary, the use of large FOVs when not required raises questions. There might not be enough awareness among clinicians about the dose-saving potential of smaller FOVs, or clinicians might habitually rely on the manufacturer-recommended default settings. Additionally, device limitations may also have played a role.

Large variation in exposure parameters was also detected between clinicians within the same diagnostic scenario. This may be due to the lack of concrete guidelines that specify settings for specific diagnostic scenarios. Moreover, some CBCT systems abstract certain exposure parameters, such as voxel size and exposure time, into presets, possibly limiting the degree of operator control. Furthermore, the large variance in reported exposure time could be attributed to confusion between scan time and exposure time, which differ in modern CBCT devices due to the use of pulsed exposures. Additionally, some participants reported using older systems with limited options for adjusting parameters such as FOV. These limitations highlight the need to phase out outdated equipment in favour of modern, configurable, and dose-optimized systems, if economically feasible.

For young patients, operators predominantly decreased exposure parameters, with operators choosing to reduce the FOV, lower the mA, shorten the exposure time, and lower the kV. Reducing kV is not supported by evidence in the literature, as an mAs (product of tube current and exposure time) reduction is both more dose-efficient and recommended for paediatric imaging.^21^

For heavier-built patients, the opposite is observed. Respondents reported raising the mA, kV, exposure time, and to a lesser degree, the FOV. Research has shown that a higher body mass index (BMI) does not negatively impact image quality.^22^ However, given the rapid advancements in CBCT technology, image quality has drastically improved at lower doses. BMI may now exert a greater influence on image quality due to advancements in low-dose imaging technology.

The use of written protocols or manufacturer-recommended settings showed no noticeable influence on exposure parameters, except for FOV area, which increased with more frequent reliance on manufacturer-recommended settings. This underscores the importance of critically evaluating the selected FOV, particularly when using manufacturer-recommended settings. Other studies, however, have shown significant dose reductions through custom exposure protocols.^6^^,^^21^^,^^23^ This suggests that the effective dose reduction depends less on the presence of a protocol, and more on how deliberately it is designed with dose optimization and the ALADAIP principle in mind. Due to the significant variability in the features of CBCT systems, clinicians must be familiar with their device’s specifications in order to select or create an appropriate protocol.^24^

CBCT requires more careful selection of exposure parameters compared to 2D imaging. Therefore, some clinics have created written protocols specifying exposure settings for different clinical scenarios. Larger clinics with multiple CBCT operators might employ more of these protocols. However, in this study, no difference was found between the number of written protocols and operators, suggesting that larger clinics do not necessarily employ more protocols, despite having multiple operators.

While the adoption of DRLs is still lacking, ensuring the DAP is recorded is a crucial first step. Fortunately, the majority of participants reported recording the DAP after each scan. This is important for the application of available DRLs, and more importantly, the establishment of new DRLs.

It is important to mention the limitations of this study. First, the surveyed participants were all members of the EADMFR, which means they may be more interested in radiology than the average dental practitioner. This could positively skew the results, as these participants might be more actively involved in dose optimization. Furthermore, it is plausible that some of the participants are also active in university hospitals, which makes it difficult to extrapolate the findings in this study to the general practice. The low response rate could have added a further degree of bias, as respondents with detailed knowledge of exposure settings are more likely to finish the survey, which may have biased the results towards lower exposure settings. Second, the survey was administered 2 years ago. CBCT exposure practices may have evolved since then, which could limit the applicability of the findings. Additionally, the survey allowed participants to provide open-ended responses for some questions, such as exposure time. This introduced ambiguity, requiring manual correction during data analysis. A larger number of responses came from the same countries, while from other countries, no responses were received (Figure 1), causing bias in our results.

Future studies should also include non-EADMFR dental practitioners using CBCT to provide a more complete image of CBCT usage. Moreover, collecting the data in a prospective manner could ensure uniformity of data, largely removing the need for manual correction. In addition, collecting the mAs value of a scan, instead of the exposure time and tube current separately, would have provided more insight into exposure practices. Filtration is another important aspect that plays a role in the effective dose a patient receives. For this study, only tube voltage values were collected; however, the resulting beam energy is also determined by the amount of filtration used. Differences in filtration between CBCT systems lead to variations in effective dose and image quality. The current study did not take this into account, as manufacturers do not always state the exact amount of filtration used, making it difficult for participants to provide this information. In addition to filtration, differences in exposure parameters could also partially be attributed to other hardware differences, such as X-ray detector sensitivity, between different CBCT systems.

The last general European guidelines on dental CBCT usage date from 2012 by the SEDENTEXCT project.^2^ While these guidelines provide a solid, evidence-based foundation, they were supposed to be updated every 5 years. Unfortunately, the European Union has not taken steps to realize this. Since 2012, several national guidelines on the use of CBCT in certain subfields of dentistry have been introduced. This led to a fragmentation in knowledge, which might have contributed to the lack of uniformity in clinical practice we encountered. The European Society of Endodontology has published a position paper on the use of CBCT in endodontics.^25^ It would be highly commendable if other European scientific societies, such as the EADMFR or those in the fields of implantology and orthodontics, were to follow this example. In doing this, they should not only address the indications for CBCT usage, but also provide concrete guidance on the selection of exposure parameters required for the diagnostic objective, taking into account the physical characteristics of the patient.

In conclusion, there is a lack of consistency in CBCT exposure practices across Europe, with large variations observed between operators. Across different diagnostic scenarios, operators predominantly adjusting voxel size and FOV. For patients with different physical characteristics, not all respondents opted to change their settings, emphasizing the need for updated, dose-optimized, indication-oriented, and patient-specific guidelines. Outdated equipment should be phased out, if economically viable, as options to adjust exposure parameters may be limited. Manufacturer-recommended FOV settings result in larger field sizes and therefore require critical evaluation. Written protocols are not necessarily more prevalent when there are multiple CBCT operators in one clinic. While DAP registration is widely adopted (90%), establishing indication-oriented DRLs remains critical. New guidelines guided by the ALADAIP principle could help standardize the selection of CBCT settings, reducing variation between clinicians and enhancing the uniformity of care.

## Supplementary Material

twaf066_Supplementary_Data
