# Supplementary material for: Dose optimization in CBCT in dentistry: a survey among EADMFR members
Source: Dentomaxillofac Radiol. 2025 Sep 24;55(1):43–51. doi: 10.1093/dmfr/twaf066 (PMC12796631; doi:10.1093/dmfr/twaf066)
Supplement: twaf066_Supplementary_Data [file twaf066_supplementary_data.zip › Supplement1-3.docx]

## Supplement 1

Table 1. Influence of the number of settings used, on the number of active operators.

| Number of CBCT-settings in use | Number of operators |
| --- | --- |
| 1 - 5 | 3.07±2.09 |
| 6 - 10 | 2.78±1.73 |
| 11 - 15 | 4.00±2.94 |
| more than 20 | 6.60±5.50 |

## Supplement 2

Table 2. Influence of the usage of written protocols on exposure parameters.

| My clinic uses written protocols specifying different settings for different diagnostic questions | Tube current (mA) | Tube voltage (kV) | Voxel size (mm) | Exposure time (s) | Field of view area (cm^2^) |
| --- | --- | --- | --- | --- | --- |
| Never | 6.69±2.64 | 97.05±11.90 | 0.17±0.06 | 10.13 ± 5.85 | 37.15±10.20 |
| Sometimes | 11.00±0.00 | 90.00±0.00 | 0.17±0.04 | 18.80±0.00 | 47.62±23.16 |
| About half of the time | 8.92±2.32 | 90.00±0.00 | 0.25±0.05 | 10.17±2.76 | 25.06±3.73 |
| Most of the time | 6.83±2.16 | 93.39±7.07 | 0.15±0.05 | 12.40±4.42 | 35.94±20.85 |
| Always | 7.61±3.07 | 92.78±10.26 | 0.18±0.07 | 11.71±8.52 | 34.21±18.64 |

## Supplement 3

Table 3. Influence of the usage of manufacturer-recommended settings on exposure parameters.

| My clinic uses CBCT settings recommended by the manufacturer | Tube current (mA) | Tube voltage (kV) | Voxel size (mm) | Exposure time (s) | FOV area (cm^2^)* |
| --- | --- | --- | --- | --- | --- |
| Never | 6.17±2.13 | 91.25±8.20 | 0.18±0.08 | 11.31±4.02 | 25.42±6.60 |
| Sometimes | 7.86±3.04 | 93.67±7.30 | 0.16±0.07 | 11.13±5.16 | 24.27±7.52 |
| About half of the time | 6.14±2.44 | 92.50±8.80 | 0.20±0.06 | 9.74±5.62 | 30.50±6.60 |
| Most of the time | 7.77±2.68 | 97.29±8.85 | 0.16±0.06 | 10.90±5.57 | 38.49±19.21 |
| Always | 8.57±3.40 | 91.39±12.71 | 0.19±0.07 | 15.35±10.30 | 53.79±22.11 |

* p<0.05
