# Supplementary material for: Dose optimization in CBCT in dentistry: a survey among EADMFR members
Source: Dentomaxillofac Radiol. 2025 Sep 24;55(1):43–51. doi: 10.1093/dmfr/twaf066 (PMC12796631; doi:10.1093/dmfr/twaf066)
Supplement: twaf066_Supplementary_Data [file twaf066_supplementary_data.zip › Supplement 4.pdf]

Supplement 4

VU VRIJE UNIVERSITEIT AMSTERDAM

Dear colleague,

Thank you for participating in this survey about CBCT exposure settings. The purpose of this survey is to get an inventory of current CBCT practices and optimization. The survey will start with 4 fictive cases followed by eight general questions. This research has been made possible with the guidance and support of dr. Erwin Berkhout, the president of European Academy of DentoMaxilloFacial Radiology (EADMFR) and dr. Reinier Hoogeveen, chair of the Committee of Selection Criteria and Radiation Protection of the EADMFR. Your participation is completely voluntary. If you have any questions, comments or difficulties, please contact m.a.martoidjojo@student.acta.nl.

1. I consent to the collection and use of my data in the manner and for the purposes stated in the information letter.

- ○ Yes
- ○ No

2. Please fill in the unique personal code that was stated in the e-mail with the link to this survey

3. Case 1 of 4: A 14-year-old girl is referred to you for CBCT scanning for impaction of upper left canine and possible resorption of upper left lateral incisor.

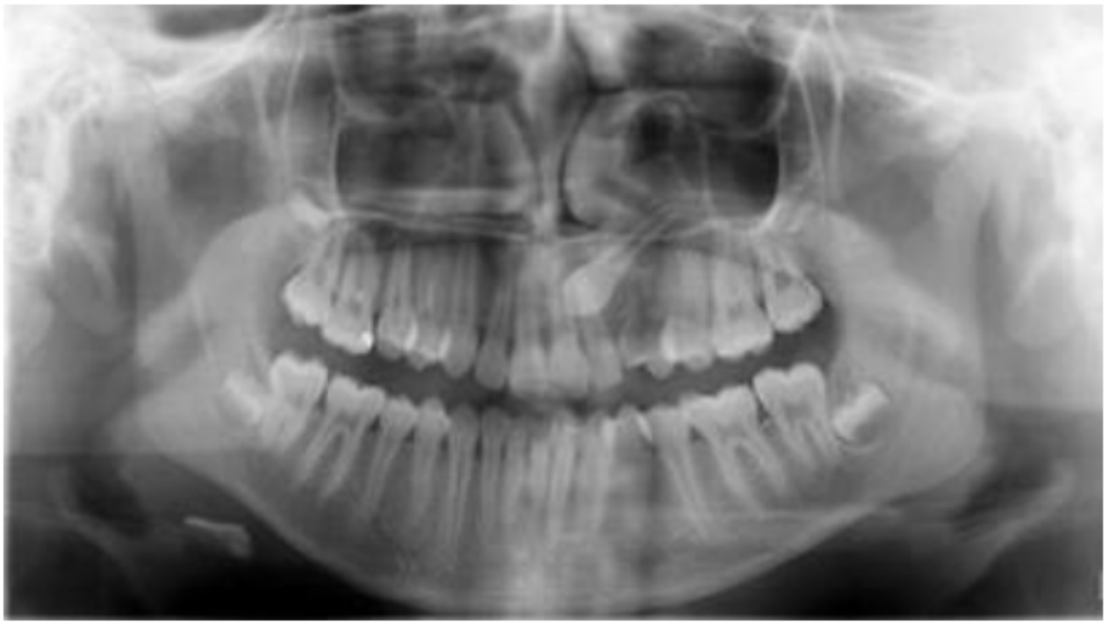

a) Please specify the make and model of the CBCT unit you would deploy

b) Which field of view (FOV) would you use? (cm x cm)

c) Which tube current (mA) would you use?

d) Which tube voltage (kV) would you use?

e) Which voxel size would you choose? (mm)

f) Which exposure time would you use? (sec)

4. Case 2 of 4 A 50 year old male is referred to you for CBCT-scanning of the area of the left lower molar for single tooth implant planning.

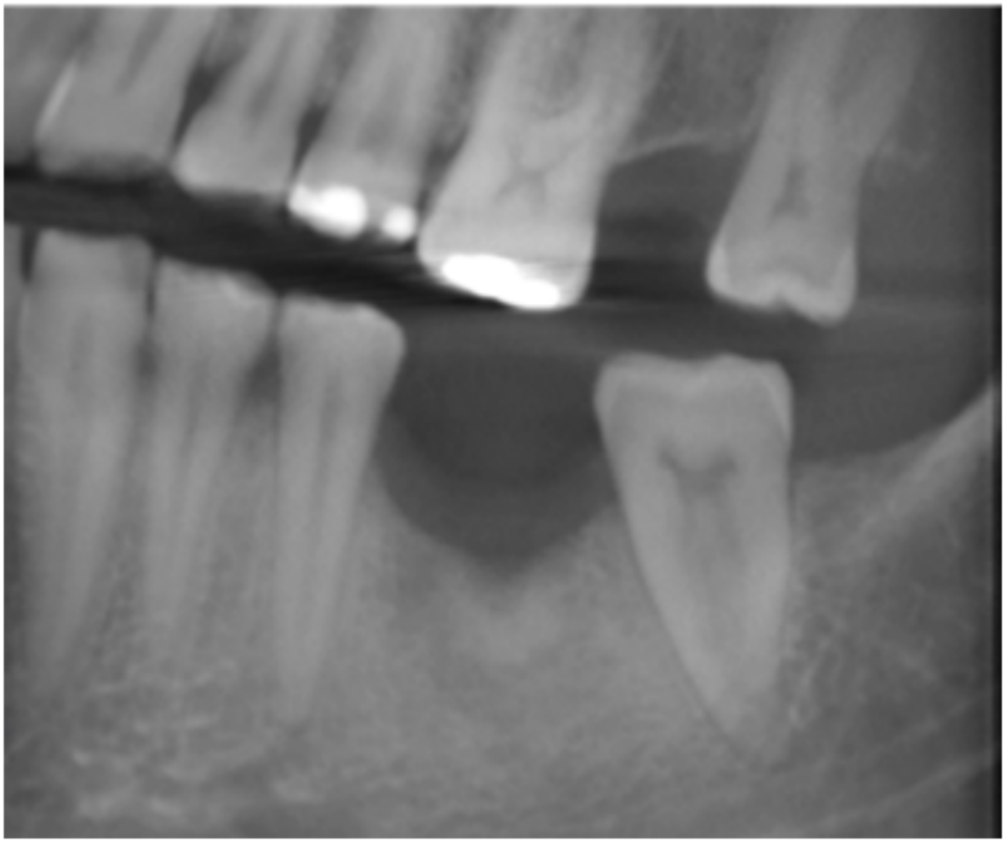

a) Please specify the make and model of the CBCT unit you would deploy

b) Which field of view (FOV) would you use? (cm x cm)

c) Which tube current (mA) would you use?

d) Which tube voltage (kV) would you use?

e) Which voxel size would you choose? (mm)

f) Which exposure time would you use? (sec)

5. Case 3 of 4 A 50-year-old woman is referred to you for CBCT-scanning of the upper left first molar for detection of a possible vertical root fracture.

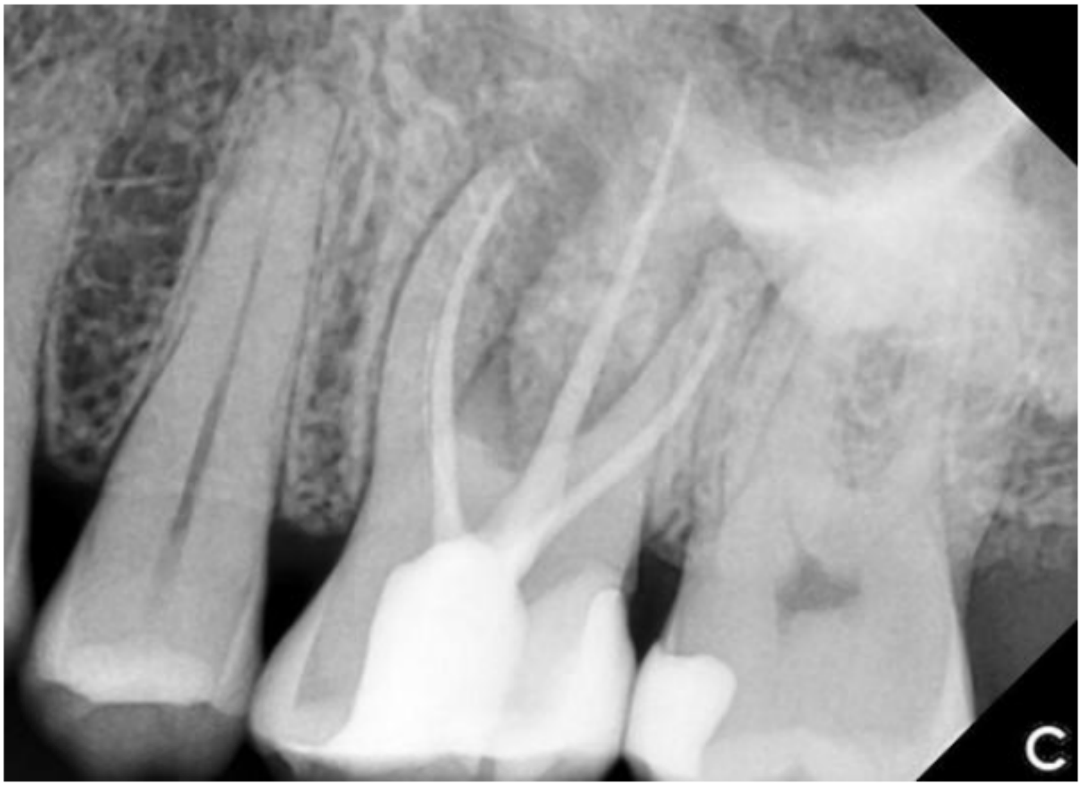

a) Please specify the make and model of the CBCT unit you would deploy

b) Which field of view (FOV) would you use? (cm x cm)

c) Which tube current (mA) would you use?

d) Which tube voltage (kV) would you use?

e) Which voxel size would you choose? (mm)

f) Which exposure time would you use? (sec)

6. Case 4 of 4 A 65-year-old male was referred to you for CBCT-scanning for implant planning for a fixed bridge construction on 4 or 6 implants in the maxilla.

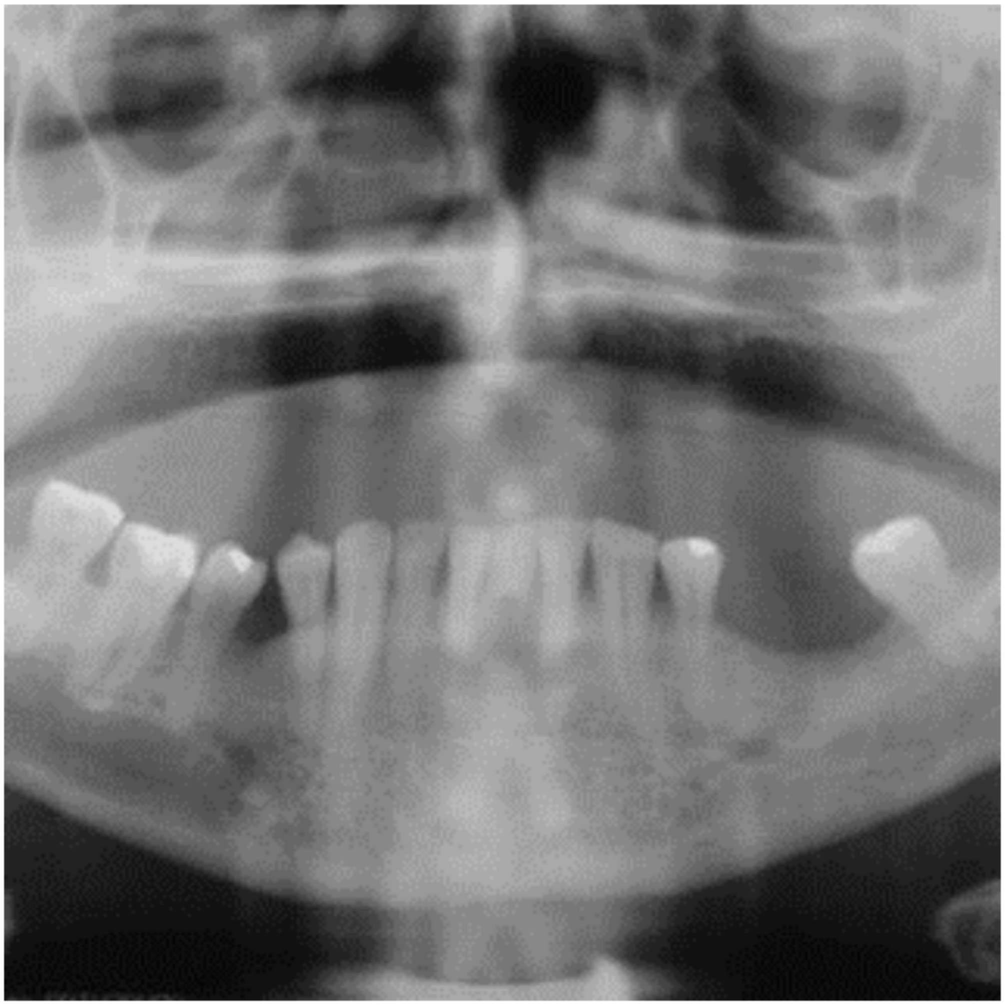

a) Please specify the make and model of the CBCT unit you would deploy

b) Which field of view (FOV) would you use? (cm x cm)

c) Which tube current (mA) would you use?

d) Which tube voltage (kV) would you use?

e) Which voxel size would you choose? (mm)

f) Which exposure time would you use? (sec)

7. Does the age of the patient influence your CBCT settings?
If yes, for a young patient: which factor would you adjust (multiple answers possible)

- ☐ mA higher
- ☐ mA lower
- ☐ kV higher
- ☐ kV lower
- ☐ Field of View size larger
- ☐ Field of view size smaller
- ☐ Voxel size larger
- ☐ Voxel size smaller
- ☐ Exposure time shorter
- ☐ Exposure time longer
- ☐ No, age does not influence my CBCT settings

8. Are physical characteristics of the patient (heavily built or fragile) influencing your CBCT settings?
a. If yes, for a heavily built patient: which factor would you adjust (multiple answers possible)

- ☐ mA higher
- ☐ mA lower
- ☐ kV higer
- ☐ kV lower
- ☐ Field of view size larger
- ☐ Field of view size smaller
- ☐ Voxel size larger
- ☐ Vozel size smaller
- ☐ Exposure time shorter
- ☐ Exposure time longer
- ☐ No, physical characteristics of the patient do not influence my CBCT settings

9. Is the Dose Area Product (DAP) registered for CBCT scans performed in your clinic?

- ○ Yes
- ○ No

10. How many CBCT operators are active in your clinic? (If one: proceed with question 12. If more, proceed with question 11)

[ ]

11. If your clinic has multiple CBCT operators, do they use the same settings in case of identical diagnostic question and patient characteristics?

	Never	Sometimes	Most of the time	Always
	0	1	2	3	4
Click to write Choice 1				

12. My clinic uses written protocols specifying different settings for different diagnostic questions

	Never	Sometimes	Most of the time	Always
	0	1	2	3	4
Click to write Choice 1				

13. My clinic uses the CBCT setting recommended by the manufacturer

	Never	Sometimes	Most of the time	Always
	0	1	2	3	4
Click to write Choice 1				

14. How many different different CBCT-settings are being used in your clinic?

- ○ 1 - 5
- ○ 6 - 10
- ○ 11 - 15
- ○ 16 - 20
- ○ more than 20
